# Nutrition Care after Discharge from Hospital: An Exploratory Analysis from the More-2-Eat Study

**DOI:** 10.3390/healthcare6010009

**Published:** 2018-01-20

**Authors:** Celia Laur, Lori Curtis, Joel Dubin, Tara McNicholl, Renata Valaitis, Pauline Douglas, Jack Bell, Paule Bernier, Heather Keller

**Affiliations:** 1 Faculty of Applied Health Sciences, University of Waterloo, Waterloo, ON N2L 3G1, Canada; cvlaur@uwaterloo.ca (C.L.); mcnicht@mcmaster.ca (T.M.); rfvalaitis@uwaterloo.ca (R.V.); 2 NNEdPro Global Centre for Nutrition and Health, Cambridge CB4 0WS, UK; pl.douglas@ulster.ac.uk; 3 Department of Economics, University of Waterloo, Waterloo, ON N2L 3G1, Canada; ljcurtis@uwaterloo.ca; 4 Department of Statistics and Actuarial Science, School of Public Health and Health Systems, University of Waterloo, Waterloo, ON N2L 3G1, Canada; jdubin@uwaterloo.ca; 5 Nutrition Innovation Centre for Food and Health (NICHE), Ulster University, Coleraine BT52 1SA, Northern Ireland; 6 School of Human Movement and Nutrition Sciences, The University of Queensland, St. Lucia 4072, QLD, Australia; Jack.Bell@health.qld.gov.au; 7 The Prince Charles Hospital, Allied Health, Chermside 4032, QLD, Australia; 8 Ordre professionnel des diététistes du Québec, Montreal, QC H3A 1B9, Canada; pbernier@opdq.org; 9 Schlegel-University of Waterloo Research Institute for Aging, Waterloo, ON N2G 0E2, Canada

**Keywords:** hospital, discharge, education, oral nutritional supplement, malnutrition

## Abstract

Many patients leave hospital in poor nutritional states, yet little is known about the post-discharge nutrition care in which patients are engaged. This study describes the nutrition-care activities 30-days post-discharge reported by patients and what covariates are associated with these activities. Quasi-randomly selected patients recruited from 5 medical units across Canada (*n* = 513) consented to 30-days post-discharge data collection with 48.5% (*n* = 249) completing the telephone interview. Use of nutrition care post-discharge was reported and bivariate analysis completed with relevant covariates for the two most frequently reported activities, following recommendations post-discharge or use of oral nutritional supplements (ONS). A total of 42% (*n* = 110) received nutrition recommendations at hospital discharge, with 65% (*n* = 71/110) of these participants following those recommendations; 26.5% (*n* = 66) were taking ONS after hospitalization. Participants who followed recommendations were more likely to report following a special diet (*p* = 0.002), different from before their hospitalization (*p* = 0.008), compared to those who received recommendations, but reported not following them. Patients taking ONS were more likely to be at nutrition risk (*p* < 0.0001), malnourished (*p* = 0.0006), taking ONS in hospital (*p* = 0.01), had a lower HGS (*p* = 0.0013; males only), and less likely to believe they were eating enough to meet their body’s needs (*p* = 0.005). This analysis provides new insights on nutrition-care post-discharge.

## 1. Introduction

There are continued pressures for early hospital discharge as soon as patients are medically stable [1]. Between 20% and 45% of patients admitted to hospital are malnourished, and many leave in the same nutritional state [2,3]. One study found that 63% of patients who stayed at least 7 days in hospital left in the same nutritional state as they were admitted, and around 20% deteriorated [4]. Between 50–80% of hospital patients report eating difficulties [5,6], and 30–70% report poor appetite [6,7,8], and these challenges may continue post-discharge. Interventions that promote good nutrition and address nutritional deficiencies are needed pre, during, and post-hospitalization to decrease a variety of complications, including mortality and readmission [9,10,11,12]. When nutrition risk is present at admission, it means that the issue has developed in the community. Appropriate discharge planning is a key mechanism to connect those malnourished or at risk patients with appropriate resources in the community. 

Approximately a quarter of patients report weight loss 30-days after discharge, which was associated with being on a “special” diet (also called a therapeutic diet) and reporting a fair/poor appetite [13]. This same study found that only 11% of participants reported consulting a dietitian post-discharge and this was associated with severe malnutrition, weight loss after discharge, comorbidity, and having seen a dietitian in hospital [14]. This research suggests need for nutrition care post-discharge, but potential gaps as well. Little is known regarding whether or not patients follow nutritional recommendations provided in hospital or what other strategies are used at home to support their nutritional status [15]. 

Strategies to improve post-discharge nutrition-care may include referral to a community dietitian [16,17,18] either by having the patient attend a clinic or having the dietitian visit the patient at home [18,19]; use of oral nutritional supplements (ONS) when needed [20,21]; a combination of dietetic counseling and ONS [22,23,24]; meal delivery programs [25,26,27,28]; or education and recommendations provided in hospital prior to discharge, among others. Several of these interventions have been found to improve body weight and self-reported food intake, however many studies lack rigor [21]. A greater understanding of who accesses or follows through on currently available resources is needed [27]. Referral to a dietitian is encouraged, including through a home visit or as part of a discharge liaison team [18,19], but access to a dietitian is not always possible and other methods of nutritional follow-up post-hospitalization should be considered. 

Education or nutrition information is provided for some patients at hospital discharge, but improvement is needed [12]. There are many barriers to nutritional counseling and to the adequate delivery of this service, such as patients being discharged too quickly before education could be provided, a lack of knowledge or interest from patients or clinicians in discussing nutrition [29], lack of health literacy, the patient’s previous healthcare experiences, as well as their health status at discharge [30]. One study reported that almost all of hospital case managers thought nutrition related diseases and risk factors (e.g., swallowing problems, poor appetite) strongly influenced discharge planning, yet dietitians were not seen as important, and were rarely consulted in discharge planning [31]. Little is known about the effect of the nutrition information provided at discharge, or who adheres to that information. Use of various types of ONS after hospital discharge [20,32,33] is another common strategy as these products have been shown to enhance patient recovery including reducing (re)admissions [34], and increasing dietary intake [20], while remaining cost effective [35,36].

To provide adequate nutrition follow-up after hospitalization, nutritional needs and risk must be identified and the appropriate treatment provided in hospital. The purpose of this investigation was to describe the nutrition-care activities that patients engaged in 30-days after discharge, and specifically determine covariates associated with the two most common activities, following nutrition recommendations provided in hospital, and/or using ONS. 

## 2. Materials and Methods

The Integrated Nutrition Pathway for Acute Care (INPAC) is an evidence and consensus based algorithm for the identification, prevention, treatment, and monitoring of malnutrition in hospital [37]. After diagnosis and treatment, INPAC recognizes the importance of considering nutrition in the discharge process to allow the support to continue as patients return to the community. The More-2-Eat (M2E) study was developed to evaluate the implementation of INPAC in five Canadian hospitals. After one year, the M2E hospitals had each implemented a unique nutrition screening and assessment triaging process that allowed all patients to receive the appropriate nutrition-care in hospital [38]. Some sites started to address the need for improved nutrition support after discharge. 

### 2.1. Ethics

Ethical approval for M2E was obtained from the University of Waterloo Research Ethics Board (ORE #20590) and from the ethics committees at each of the five participating hospitals (Niagara Health Ethics Board, Ottawa Health Science Network Research Ethics Board, Health Research Ethics Board of the University of Alberta, Regina Qu’Appelle Health Region Research Ethics Board, Concordia Research Ethics Committee). Data collection directly from patients or staff required informed written consent, which was attained prior to data collection and was specific to the collection of 30-day follow-up data. 

### 2.2. Sample and Participants

The study populations were all patients on the chosen medical unit at the: Royal Alexandra Hospital (Edmonton, AB, Canada), Regina Pasqua Hospital (Regina, SK, Canada), Concordia Hospital (Winnipeg, MB, Canada), Niagara Health System, General Site (Niagara Falls, ON, Canada), and Ottawa Hospital (Ottawa, ON, Canada). Data on nutrition, frailty (hand grip strength; HGS), disability, quality of life, and food intake was collected on a subset of patients recruited in M2E each month for 15-months (referred to as “detailed patient data”; *n* = 1250). These data were used to understand how strategies to implement INPAC started to affect the quality of care, such as reducing mealtime barriers in hospital for patients. At three time points (baseline, months 5/6, and 11/12 of implementation) detailed data collection patients recruited also consented to data collection 30-days after their discharge (referred to as “30-day data”). Telephone surveys were used to collect the 30-day data. 

The eligibility criteria for patients were driven by the primary objectives of the M2E study: (1) no cognitive impairment as per admission assessment by nursing; (2) spoke English (or French at Ottawa site); (3) consumed an oral diet, but supplemental enteral or parenteral nutrition were allowed; (4) admitted from the community and likely to return home to the community; and (5) provided written consent to participate. The sampling design was single stage and participants were chosen through a quasi-random sampling process, with additional details provided elsewhere [38]. 

### 2.3. Data Collection and Measures

#### 2.3.1. In-Hospital Data Collection

A clinical dietitian or nurse was seconded from each hospital for M2E data collection. The variables from the detailed patient data used within this analysis included: age, sex, primary admission diagnosis (categorized into the six most commonly reported diagnoses), nutrition risk, nutritional status, length of stay (LOS), HGS, use of ONS in hospital, patient perception of importance of food intake on recovery, patient perception of staff’s view on the importance of food intake, and barriers to food intake at a single meal. 

Nutrition risk was measured using the Canadian Nutrition Screening Tool (CNST) [39]; two questions on 6-month weight loss and food intake over the past week are asked of patients or their proxy. Nutritional status was determined using the subjective global assessment (SGA A: well-nourished, SGA B: mild/moderately malnourished, SGA C: severely malnourished) [40], with SGA B and C amalgamated for analysis. SGA is considered a diagnostic tool including food intake, function, weight change, and body composition, and is completed by a trained clinician. To measure HGS, training was provided on the Southampton protocol for HGS using a Jamar hydraulic hand dynamometer J0001057 [41]. The Mealtime Audit Tool was used to measure barriers to food intake while in hospital at a single meal [42]. The My Meal Intake Tool was used to assess food intake at a single meal using a scale of 0%, 25%, 50%, 75%, or 100% of their food/beverages [43]. Patient perception scores regarding the impact of food on recovery and their perception of staff’s views on the importance of food intake were measured on a scale of 1 (low), 10 (high). A cut point of 7+ was used to categorize responses.

#### 2.3.2. Data Collection 30-Days after Discharge

Patients recruited for the 30-day data collection provided contact information, including a proxy (a family member or friend to be contacted if the patient could not be reached). These participants were contacted 30-days after the discharge date by university researchers, with up to five calls attempted to reach participants. When proxy contact information was available and the patient could not be reached, the proxy was called up to three times. If a person who answered the phone reported a patient was unavailable due to death, a move to a residential facility, had returned to hospital, or other, this was recorded. All data were collected by self-report or proxy-report.

Weight change included options of weight gain of >5 lbs, loss of >5 lbs, no change (e.g., <5 pounds), or did not know. For weight gain or loss, the participant was asked whether or not this was intentional, and for weight loss whether or not this was related to changes in edema (asked as fluid loss for participants). Appetite was rated and categorized as Very Good/Good, and Fair/Poor for analysis. Questions also explored whether the patient was: avoiding certain foods, following a special diet, having a diet different than before hospitalization, receiving meals from a program or service, if they received information about diet or community services to support recovery after discharge, if they followed those recommendations, and if they took meal replacements or supplements (ONS), all with a dichotomous response option (Yes/No). 

The use of other health services specific to nutrition-care was also reported (Yes/No): whether they saw a doctor; if the doctor asked about nutrition or body weight; if they followed the recommendations provided by the doctor; whether they had seen a dietitian (and where); having seen another healthcare provider (and which one); having an emergency room visit since discharge (and how many times); having a health clinic/emergency clinic visit since discharge (and how many times); and being readmitted since discharge (and how many times). For analysis, emergency room visits and health/emergency clinic visits were combined due to low prevalence. Food related activities of daily living included eating one or more meals with someone, where responses were categorized as Never or Rarely/Sometimes vs. Often/Almost Always for analysis. The participant was also asked their perception on how they were eating, categorized as more than enough/enough vs. not enough for analysis, and to rate their general health and nutrition health (Excellent/Very Good/Good, and Fair/Poor). 

Open-ended questions asked for specifics when avoiding certain foods or following a special diet, how the diet differed from before hospitalization, who helped with preparing meals, details when a meal program or service was used, the specific hospital recommendations that the patient followed, the frequency and type of ONS used, any new services provided after a conversation with their doctor or dietitian, if the patient did anything to support their food intake after discharge, and if anything else could have been done by the hospital to support their food intake and recovery after hospitalization.

### 2.4. Data Analysis

Sample characteristics and nutrition-care variables post-discharge were analyzed descriptively. Analyses were completed to determine if respondents (those who completed 30-day data collection) were representative of those who consented to participate in this follow-up. Those who died/hospitalized/moved were categorized as non-completers. Comparisons were made on demographics (sex, age, site), diagnoses, LOS, and nutrition-care variables to determine potential bias in the respondents. 

Due to the low prevalence of most nutrition-care variables (e.g., reported seeing a dietitian), bivariate analyses to characterize users of services were only completed for those activities where more than 25% of the sample reported the activity. This included only two care activities: following nutrition recommendations provided by a clinician in the hospital, and use of ONS post-discharge. Bivariate analyses were completed for covariates of interest hypothesized to predict these care activities. Chi-square tests were used for associations of categorical variables such as sex, nutrition risk, appetite, etc. *t*-tests were used for associations with continuous variables including age, LOS, and HGS. Due to the number of comparisons made, to reduce type I error the significance level was set at *p* < 0.01 for this exploratory analysis. Statistical analysis was completed using SAS Studio 9.4 for Windows. The answers to the open-ended question about following dietary recommendations were grouped into content themes; for frequency of ONS use, responses were separated into timeframes (daily, occasionally etc.). 

## 3. Results

### 3.1. Overall Sample

A total of 513 participants were recruited for 30-day follow-up after discharge from hospital. Of these, 48.5% (*n* = 249) completed this telephone follow-up. Non-respondents were: proxy completion (*n* = 16); patient death (*n* = 7); moved to a residential facility (*n* = 2); in hospital at time of interview (*n* = 19); declined (*n* = 60); or did not answer the phone (*n* = 160). Respondents and non-respondents were not significantly different on any demographic or nutrition-care variables. Thirty-day respondents had a significantly (*t* = −2.71, *p* = 0.007) shorter LOS (9.1 days SD 7.21) as compared to those who did not participate (10.5 days SD 9.39), and were more likely to have an ‘other’ diagnosis (*t* = −2.72, *p* = 0.007; 17.5% overall and 12.1% in 30-day participants), or a neurological diagnosis (8.6% overall and 11.7% in 30-day participants). Participant characteristics, in hospital nutrition-care (Table 1), and post-discharge nutrition indicators and care activities (Table 2) are provided. 

Of the 30-day follow-up participants, 40% (*n* = 101) were male; the mean age was 70 (SD 14.7); 21% (*n* = 53) were at nutrition risk; and 14.5% (*n* = 36) were SGA B or C (malnourished). At discharge, 44% (*n* = 110) received information about food intake/community food related services, and of those 65% (*n* = 71/110) reported following those recommendations. Within 30-days of discharge, 19% (*n* = 47/249) reported losing weight, with 81% (*n* = 38/47) reporting that this was unintentional; 40% (*n* = 19/47) of participants reporting weight loss had received nutrition information in hospital. About a third (*n* = 84) of patients reported never/sometimes eating with others. In hospital, 8% (*n* = 20) were taking ONS, while 26.5% (*n* = 66) took ONS after discharge. Only 6.8% (*n* = 17) reported having consulted a dietitian since discharge.

After hospitalization, 42% (*n* = 105) of participants reported following a special diet, with main dietary changes including reduction of salt, sugar, fat, consumption of specific foods, to follow a diabetic diet, and generally trying to eat healthier. Just under 24% (*n* = 59) of patients said their diet was different than before hospitalization, with the most common reasons being that their diet had improved, decreased salt/sodium, increased protein, was an overall healthier diet, or it was different because they were not eating well before hospitalization. Only 5.6% (*n* = 14) reported using a meal program or service, with the most common services listed as having meals delivered to the home, home care provision of a meal, or a general meal program for older adults. When asked if the patient did anything else to support their food intake after discharge, responses (*n* = 143) included receiving help with grocery shopping and/or cooking, or receiving prepared food from friends or family. With respect to asking about further hospital support, 73% (*n* = 181) reported that nothing else could have been done to improve their nutrition, 9% (*n* = 22) were not sure, and 18% (*n* = 46) provided suggestions for improvement. Typical comments for improvement were: the desire to see a dietitian during their stay or at discharge, for more information to be provided about nutrition at discharge, complaints about the food while in hospital, and feedback regarding improvements to general hospital care. 

### 3.2. Self-Reported Use of Discharge Nutrition Information 

A common post-hospitalization treatment strategy is the provision of education or nutrition information at discharge. Of the 42% (*n* = 110) who reported receiving nutrition recommendations at discharge, 65% (*n* = 71/110) followed the information provided. It is unknown who provided the information. Participants who received and used this information (*n* = 71) were more likely to report following a special diet (67.6% *n* = 48, X^2^ = 9.55, *p* = 0.002), or a diet different than before hospitalization (40.9%, *n* = 29, X^2^ = 7.13, *p* = 0.008), compared to those who said they did not follow the information they were provided (*n* = 38; *n* = 1 did not know if they followed the information). Those following the recommendations provided also perceived their nutritional health to be better than those who did not follow recommendations provided (fair/poor 12.7% vs. 36.8% respectively, X^2^ = 8.68, *p* = 0.003). Details provided in Table 3. 

The majority of participants (*n* = 71) who responded to the open-ended questions regarding following in-hospital recommendations described eating or avoiding specific foods such as general healthy eating tips or ways to eat more protein. Other recommendations followed included: seeking support from a healthcare professional/service such as home care or a dietitian; use of community service such as a home meal delivery program; when or how to eat, such as eating small meals, eating when hungry, or chewing enough to avoid choking; dietary recommendations for specific conditions, including diabetes; use of ONS or vitamin supplements; and advice for drug-nutrient interactions. 

### 3.3. Self-Reported Use of Oral Nutritional Supplements after Hospitalization 

Table 4 includes a comparison of 30-day follow-up participants who did and did not report using ONS, a common post-hospitalization treatment strategy. ONS was used by 26.5% (*n* = 66) of the sample post-hospitalization (*n* = 1 did not know). It is unknown if patients were following a recommendation from the hospital, or started ONS use on their own. Use ranged from several times per day, daily, a few times a week, to occasionally. Those taking ONS were more likely to be at nutritional risk (39.4%, *n* = 26; X^2^ = 17.38, *p* < 0.0001) or malnourished (SGA B or C) (27.3%, *n* = 18; X^2^ = 11.79, *p* = 0.0006) while in hospital, to have received ONS in hospital (15.2% *n* = 10, X^2^ = 6.09, *p* = 0.01), and be males with a lower in-hospital HGS (mean 23.8kg, *n* = 24, *t* = −3.33, *p* = 0.0013) as compared to those not taking ONS after discharge. Those taking ONS were also more likely to have a hospital-admission diagnosis of a respiratory nature (X^2^ = 17.65, *p* = 0.0072). Participants taking ONS were also less likely to believe they were eating enough to meet their body’s needs (80.3% stated eating enough, *n* = 167, X^2^ = 7.95, *p* = 0.005) as compared to those not reporting use of ONS (92.8%). 

## 4. Discussion

This exploratory analysis found that the most commonly reported nutrition-care activities post-discharge were following information provided in-hospital about food intake/community food related services (44% received info, and 65% followed the information), and use of ONS (26.5%). Only 6.8% of participants reported seeing a dietitian post-discharge, which is consistent with the 11% reported in a previous Canadian study [14]. Similarly, weight gain, loss, and stability were comparable to other Canadian work [14]. 

Participants who reported following the hospital dietary recommendations were more likely to be following a special diet that was different from their pre-hospitalization diet, compared to those who did not follow the recommendations. These participants were also less likely to rate their nutritional health as fair/poor. As only a few characteristics were associated with who follows the nutrition information, this could indicate that the use of this information is not limited by age, gender, or nutrition risk. Some of the recommendations provided were focused on specific conditions (diabetes control etc.) or weight loss, while others encouraged food intake, and ways to increase protein. Further information regarding specific recommendations and who followed them, would help to highlight areas for malnutrition prevention, such as encouraging adequate protein intake in older adults at home. 

It is unknown who provided the nutrition information received by patients at discharge. When needed, dietitians should provide patients with a nutritional care plan, counseling, and monitoring of nutritional status during and post-discharge. Studies by Beck and colleagues (2013, 2014), found beneficial effects of seeing a dietitian post-discharge, including adding a dietitian to a discharge liaison team [18,19]. As a specialist resource, dietitians are not always consulted or available at the time of discharge [31]. With support from the dietetics team, and within professional boundaries, an interprofessional approach can be used to promote food intake and encourage adequate nutrition, or community nutrition support [44]. A proactive nutrition culture should be encouraged among all staff to recognize that nutrition is important [44]. INPAC advocates that discharge planning include referral to community-based dietitians to continue nutritional therapy started in hospital for malnourished patients [37]. Other trained health professionals in hospital should also be involved to ensure food access is available post-discharge. For example, an occupational therapist can assess for cooking abilities, or a social worker can connect with community services, such as a home meal delivery program.

As only around 7% of patients reported consulting a dietitian after discharge, yet 72% consulted a doctor, general practitioners/family medicine are a potential avenue for referral to a community-based dietitian for those who continue to lose weight and are struggling with their nutrition. Nutrition education for physicians is lacking [45], and time is another significant barrier to providing nutrition advice [46]. Educating physicians on nutrition screening and appropriate referrals to a dietitian or nutrition service in the community is needed. Further understanding of how and why patients consult a dietitian post-discharge is an area for future research. 

With respect to ONS, there was no difference in use for those who reported weight loss or gain, even though taking these supplements was associated with nutrition risk. This lack of difference may be due to the short time frame, the variability in frequency of ONS use (from several times per day through to only occasionally), or the amount taken, and is inconsistent with experimental studies on use of these products [20,21]. This analysis is based on the first known observational study exploring ONS users post-hospitalization. Although new insights into whom is more likely to use these products (e.g., not eating well, at risk/malnourished), resulted, further work, potentially through qualitative investigations, is needed to understand use. 

These exploratory results can be used to build on the growing literature regarding outcomes for patients after hospital discharge. Understanding who currently follows through on recommendations or uses ONS provides a foundation for further work on whom to target for such nutrition-care activities. Referral to a dietitian is encouraged for malnourished patients, yet is not always feasible; other support mechanisms should be considered while a solution to this shortage of this specialized resource is put into place.

### Limitations

There are several limitations to these data, particularly due to the small sample size, however it was still generally representative of the detailed patient sample who consented for 30-day follow-up. The shorter LOS of respondents vs. non-respondents may indicate that 30-day participants were somewhat healthier than non-participants. The eligibility criteria of the detailed patient sample excluded those with cognitive impairment, and those returning to locations other than home in the community. Results can thus not be extrapolated to all medical patients. The population was also not restricted to those who were malnourished, and many of the recommendations may have been for losing weight, or specific to admission diagnosis, particularly diabetes. Participants needed to be well enough to answer the phone (and the questionnaire), able to hear the questions (several interviews were ended due to participant hearing difficulties), and available to answer the call. With calls coming from the University of Waterloo, rather than from a hospital, many calls may have been screened and left unanswered. The interviewers aimed to address this issue by leaving a message on the fourth call. In future, it may be beneficial to have follow up interviews conducted by the recruiting hospital. The number of participants who died, moved to a residential facility, or returned to hospital may not be reflective of the population. These data are based on reports from proxies (or whomever answered the phone), and does not include those who did not answer the phone. 

The open-ended question about ONS use elicited some indication of the frequency of use, however due to the small sample and large variability in responses, this should be interpreted with caution. Regarding the nutrition recommendations provided at discharge, qualitative results provide examples of the type of advice or information provided. It is unknown who provided the information, and a month after discharge, participants did not always remember what they were told, or what they did remember was vague. Some participants specified if the information was provided by a nurse or dietitian, however most did not specify or could not remember. Future investigations should collect data on type and provider of recommendations from the admitting hospital. Understanding the sources of the education, and the type of information provided is necessary to further understand how nutrition is included at discharge. Ideally, it would be beneficial to know how: (i) the patients are using this information; (ii) whether it is changing their behavior; and (iii) if it is having an impact on outcomes. Future work could also explore post-discharge ONS consumption including reason for use (recommendation from hospital or not), frequency of use, duration, amount, etc. Finally, focusing 30-day follow-up data collection on patients malnourished in hospital would help to further identify gaps in nutrition-care for these highly vulnerable patients upon their return home. 

## 5. Conclusions

This exploratory analysis provides new insights on the prevalence of nutrition-care post-discharge. Most post-discharge nutrition-care activities were low in frequency, particularly consulting a dietitian. Of all nutrition-care activities post-discharge, participants most commonly reported following nutrition recommendations provided in hospital, or using ONS. These findings provide areas for further exploration regarding nutrition-care activities and characteristics of patients more likely to receive/follow recommendations. Further work is needed to understand how to best support patients after discharge, considering patient preferences and health outcomes. 

## Figures and Tables

**Table 1:** In-hospital characteristics of 30-day follow-up participants (*n* = 249).

Demographic	All 30-Day Follow-Up Participants % (n)/Mean (SD)
*Demographics*	
Male Sex	40.1 (101)
Age (years)	70.0 (SD 14.73)
*In Hospital Variables*	
Primary Diagnosis:	
Cardiovascular	14.9 (37)
Gastrointestinal	10.8 (27)
Respiratory	33.3 (83)
Musculoskeletal	6.8 (17)
Neurological	11.7 (29)
Infection	10.4 (26)
Other	12.1 (30)
LOS (days)	9.1 (SD 7.21) [median 7]
Hand Grip Strength in hospital (kg)	
Males	30.3 (SD 11.6 *n* = 93)
Females	15.6 (SD 8.3 *n* = 138)
At nutrition risk (at admission)	21.3 (53)
SGA B or C (malnourished)	14.5 (36)
Received advanced nutrition-care strategies in hospital	35.7 (89)
Used ONS in hospital	8.0 (20) ^a^
Eating ≤50% in hospital	33.1 (82)
Barriers to food intake/hospital	
0–2 barriers	66.9 (166)
3–5 barriers	28.6 (71)
6+ barriers	4.4 (11)
Patient perceived food has a strong impact on recovery *	85.4 (210)
Patients thought that staff considered nutrition important for their recovery *	69.4 (168) ^b^

^a^ Missing *n* = 1; ^b^ Missing *n* = 6; * On a scale of 1–10, with 10 the highest; cut point of 7+ used for strong impact; SGA, Subjective Global Assessment; LOS, length of stay; ONS, Oral Nutritional Supplement; Note: Percent of population (n) is used for dichotomous variables and mean (SD) for continuous variables.

**Table 2:** Discharge characteristics of 30-day follow-up participants (*n* = 249).

Demographic	All 30-Day Follow-Up Participants % (n)
*Perceived General Health*	
Fair/poor	42.2 (105)
*Nutritional Indicators*	
Weight change since discharge	
Gain Weight	15.3 (38)
Lost Weight	18.9 (47)
Unintentional loss	80.9 (38/47)
Stayed the Same	61.9 (154)
Do not know	4 (10)
Poor Appetite	32.5 (81)
Eats enough to meet the body’s needs	89.5 (221) ^a^
Nutrition health perceived as fair/poor	22.9 (57)
*Food Related Activities*	
Following a Special Diet	42 (105)
Diet is different than before hospitalization	23.7 (59)
Never/sometimes eat with others	33.7 (84)
Who prepares the meals:	
The patient	45.4 (113)
Shared	23.3 (58)
Someone else	31.3 (78)
*General Care Activities*	
Consulted their doctor since discharge	71.9 (179) ^b^
Consulted another HCP since discharge	46.6 (116) ^b^
Readmitted or Emergency Room/Clinic Visit	19.0 (47) ^c^
*Nutrition-Care Activities*	
Consulted a dietitian since discharge	6.8 (17) ^b^
Received meals from a program or service	5.6 (14)
Took ONS since discharge	26.5 (66) ^c^
Received information about diet/community food related services at discharge	44.2 (110) ^d^
Followed through on nutrition recommendations	65.1 (71/110) ^a^

^a^ Missing *n* = 1; ^b^ Do not know = 1; ^c^ Missing *n* = 2; ^d^ Do not know = 3; HCP, Healthcare Professional; ONS, Oral Nutritional Supplement.

**Table 3:** Comparison of 30-day follow-up participants who received and used discharge nutrition information vs. those who received and did not use the information (*n* = 109 ^a^).

Demographic	30-Day Follow-Up Participants Following the Nutrition Discharge Recommendations % (n)/Mean (SD) *n* = 71 ^a^	30-Day Follow-Up Participants NOT Following the Nutrition Discharge Recommendations % (n)/Mean (SD) *n* = 38
*Demographics*		
Male	42.0 (30)	44.7 (17)
Age (years)	66.9 (SD 12.9)	68.9 (SD 15.48)
*In Hospital Variables*		
Diagnosis:		
Cardiovascular	15.5 (11)	18.4 (7)
Gastrointestinal	15.5 (11)	2.6 (1)
Respiratory	33.8 (24)	34.2 (13)
Musculoskeletal	1.4 (1)	7.9 (3)
Neurological	16.9 (12)	2.6 (1)
Infection	4.2 (3)	13.2 (5)
Other	12.7 (9)	21.1 (8)
LOS (days)	9.7 (SD 7.6)	8.0 (SD 5.0)
Hand Grip Strength in hospital (kg)		
Males	26.7	34.1
	(10.7 SD *n* = 27/44) ^b^	(9.8 SD *n* = 17/44)
Females	16.2	16.3
	(9.4 SD *n* = 41/59) ^b^	(7.5 SD *n* = 18/59)
At nutrition risk (at admission)	28.2 (20)	29.0 (11)
SGA B or C (malnourished)	21.1 (15)	15.8 (6)
Received advanced nutrition-care strategies in hospital	42.3 (30)	47.4 (18)
Used ONS in hospital	16.9 (12)	5.3 (2)
Eating ≤50% in hospital	35.2 (25)	31.6 (12)
*Nutritional Indicators Post Discharge*		
Weight change since discharge		
Gain Weight	18.3 (13)	21.1 (8)
Lost Weight	15.5 (11)	21.1 (8)
Stayed the Same	62.0 (44)	55.3 (21)
Do not know	4.2 (3)	2.6 (1)
Unintentional Weight Loss	63.6 (7/11)	87.5 (7/8)
Poor Appetite	26.8 (19)	36.8 (14)
Eats enough to meet the bodies needs	93.0 (66)	81.6 (31)
Nutrition health perceived as fair/poor	12.7 (9) *	36.8 (14)
*Food Related Activities Post Discharge*		
Following a special diet	67.6 (48) *	36.8 (14)
Diet is different than before hospitalization	40.9 (29) *	15.8 (6)
*General Care Activities Post Discharge*		
Readmitted or Emergency Room	17.5 (11) ^c^	19.8 (36)
*Nutrition-Care Activities Post Discharge*		
Consulted a dietitian since discharge	15.5 (11)	5.3 (2)

* *p* < 0.01 ^a^ Omitted one participants who ‘did not know’ if they followed the information. ^b^ Missing *n* = 3 ^c^ Missing *n* = 2. SGA, Subjective Global Assessment; LOS, length of stay; ONS, Oral Nutritional Supplement; Note: Percent of population (n) is used for dichotomous variables and mean (SD) for continuous variables.

**Table 4:** Comparison of 30-day follow-up participants and self-reported oral nutritional supplement (ONS) use after hospitalization (*n* = 248 ^a^).

Demographic	30-Day Follow-Up Participants Taking ONS after Hospitalization % (n)/Mean (SD) *n* = 66	30-Day Follow-Up Participants NOT Taking ONS after Hospitalization % (n)/Mean (SD) *n* = 182
*Demographics*		
Male Sex	39.4 (26)	41.2 (75)
Age (years)	70.4 (SD 13.04)	66.9 (182)
*In Hospital Variables*		
Diagnosis:		
Cardiovascular	10.6 (7)	16.5 (30)
Gastrointestinal	16.7 (11)	8.8 (16)
Respiratory	45.5 (30)*	29.1 (53)
Musculoskeletal	3.0 (2)	8.2 (15)
Neurological	1.5 (1)	15.4 (28)
Infection	10.7 (7)	9.9 (18)
Other	12.1 (8)	12.1 (22)
LOS (days)	10.7 (SD 7.37) ^b^	8.4 (SD 7.09)
Hand Grip Strength in hospital (kg)		
Males	23.8 **	32.6
	(8.5 SD *n* = 24/93) ^c^	(11.8 SD *n* = 69/93)
Females	14.4	16.0
	(7.6 SD *n* = 34/138) ^d^	(8.6 SD *n* = 104/138)
At nutrition risk (at admission)	39.4 (26) ***	14.8 (27)
SGA B or C (malnourished)	27.3 (18) **	9.9 (18)
Use ONS in hospital	15.2 (10) *	5.5 (10)
Eating ≤50% in hospital	39.4 (26)	30.8 (56)
*Nutritional Indicators Post Discharge*		
Weight change since discharge:		
Gain Weight	21.2 (14)	13.2 (24)
Lost Weight	18.2 (12)	19.2 (35)
Stayed the Same	57.6 (38)	63.2 (115)
Do not know	3.0 (2)	4.4 (8)
Unintentional Weight Loss	91.7 (11/12)	77.1 (27/35)
Poor Appetite	42.4 (28)	29.1 (53)
Eats enough to meet the body’s needs	80.3 (53) ^e,^*	92.8 (167)
Nutrition health perceived as fair/poor	33.3 (22)	19.2 (35)
*Food Related Activities Post Discharge*		
Never/sometimes eat with others	36.4 (24)	32.4 (59)
*General Care Activities Post Discharge*		
Readmitted or Emergency Room/Clinic	17.5 (11) ^b^	19.8 (36)
*Nutrition-Care Activities Post Discharge*		
Consulted a dietitian since discharge	3.0 (2)	8.4 (15)
Receives meals from a program or service	6.1 (4)	5.5 (10)

* *p* < 0.01, ** *p* < 0.001, *** *p* < 0.0001; ^a^ 1 participant did not know if they were taking ONS; ^b^ Missing = 3; ^c^ Missing = 8; ^d^ Missing = 9; ^e^ Missing = 2; SGA, Subjective Global Assessment; LOS, length of stay; ONS, Oral Nutritional Supplement; Note: Percent of population (n) is used for dichotomous variables and mean (SD) for continuous variables.

## References

[1] Canadian Institute for Health Information Hospital Care. https://www.cihi.ca/en/types-of-care/hospital-care.

[2] Barker L.A., Gout B.S., Crowe T.C. (2011). Hospital Malnutrition: Prevalence, Identification and Impact on Patients and the Healthcare System. Int. J. Environ Res. Public Health.

[3] Allard J.P., Keller H., Jeejeebhoy K.N., Laporte M., Duerksen D., Gramlich L., Payette H., Bernier P., Vesnaver E., Davidson B. (2016). Malnutrition at hospital admission: Contributors and effect on length of stay. A prospective cohort study from the Canadian Malnutrition Task Force. J. Parenter. Enteral Nutr..

[4] Allard J., Keller H.H., Teterina A., Jeejeebhoy K., Laporte M., Duerksen D., Gramlich L., Payette H., Bernier P., Davidson B. (2015). Factors associated with nutritional decline in hospitalised medical and surgical patients admitted for 7 d or more: A prospective cohort study. Br. J. Nutr..

[5] Westergren A., Lindholm C., Axelsson C., Ulander K. (2008). Prevalence of eating difficulties and malnutrition among persons within hospital care and special accommodations. J. Nutr. Health Aging.

[6] Westergren A., Unosson M., Ohlsson O., Lorefält B., Hallberg I.R. (2002). Eating difficulties, assisted eating and nutritional status in elderly (≥65 years) patients in hospital rehabilitation. Int. J. Nurs. Stud..

[7] Keller H.H., Allard J., Vesnaver E., Laporte M., Gramlich L., Bernier P., Davidson B., Duerksen D., Jeejeebhoy K., Payette H. (2015). Barriers to food intake in acute care hospitals: A report of the Canadian Malnutrition Task Force. J. Hum. Nutr. Diet..

[8] Kagansky N., Berner Y., Koren-Morag N., Perelman L., Knobler H., Levy S. (2005). Poor nutritional habits are predictors of poor outcome in very old hospitalized patients. Am. J. Clin. Nutr..

[9] Krumholz H.M. (2013). Post-hospital syndrome—An acquired, transient condition of generalized risk. N. Eng. J. Med..

[10] Liu L., Bopp M.M., Roberson P.K., Sullivan D.H. (2002). Undernutrition and risk of mortality in elderly patients within 1 year of hospital discharge. J. Gerontol. Ser. A Biol. Sci. Med. Sci..

[11] Ramage-Morin P.L., Gilmour H., Rotermann M. (2017). Nutritional risk, hospitalization and mortality among community-dwelling Canadians aged 65 or older. Health Rep..

[12] Tappenden K.A., Quatrara B., Parkhurst M.L., Malone A.M., Fanjiang G., Ziegler T.R. (2013). Critical role of nutrition in improving quality of care: An interdisciplinary call to action to address adult hospital malnutrition. J. Parenter. Enteral Nutr..

[13] Keller H., Laporte M., Payette H., Allard J., Bernier P., Duerksen D., Gramlich L., Jeejeebhoy K. (2017). Prevalence and predictors of weight change post discharge from hospital: A study of the Canadian Malnutrition Task Force. Eur. J. Clin. Nutr..

[14] Keller H., Payette H., Laporte M., Bernier P., Allard J., Duerksen D., Gramlich L., Jeejeebhoy K. (2017). Patient-reported dietetic care post hospital for free-living patients: A Canadian Malnutrition Task Force Study. J. Hum. Nutr. Diet..

[15] Young A.M., Mudge A.M., Banks M.D., Rogers L., Allen J., Vogler B., Isenring E. (2015). From Hospital to Home: Limited Nutritional and Functional Recovery for Older Adults. J. Frailty Aging.

[16] Munk T., Tolstrup U., Beck A.M., Holst M., Rasmussen H.H., Hovhannisyan K., Thomsen T. (2016). Individualised dietary counselling for nutritionally at-risk older patients following discharge from acute hospital to home: A systematic review and meta-analysis. J. Hum. Nutr. Diet..

[17] Hamirudin A.H., Walton K., Charlton K., Carrie A., Tapsell L., Milosavljevic M., Pang G., Potter J. (2017). Feasibility of home-based dietetic intervention to improve the nutritional status of older adults post-hospital discharge. Nutr. Diet..

[18] Beck A.M., Kjær S., Hansen B.S., Storm R.L., Thal-Jantzen K., Bitz C. (2013). Follow-up home visits with registered dietitians have a positive effect on the functional and nutritional status of geriatric medical patients after discharge: A randomized controlled trial. Clin. Rehabil..

[19] Beck A., Andersen U.T., Leedo E., Jensen L.L., Martins K., Quvang M., Rask K.Ø., Vedelspang A., Rønholt F. (2015). Does adding a dietician to the liaison team after discharge of geriatric patients improve nutritional outcome: A randomised controlled trial. Clin. Rehabil..

[20] Milne A.C., Potter J., Vivanti A., Avenell A. (2009). Protein and energy supplementation in elderly people at risk from malnutrition (review). Cochrane Database Syst. Rev..

[21] Beck A.M., Holst M., Rasmussen H.H. (2013). Oral nutritional support of older (65 years+) medical and surgical patients after discharge from hospital: Systematic review and meta-analysis of randomized controlled trials. Clin. Rehabil..

[22] Persson M., Hytter-Landahl Å., Brismar K., Cederholm T. (2007). Nutritional supplementation and dietary advice in geriatric patients at risk of malnutrition. Clin. Nutr..

[23] Neelemaat F. (2012). Post-Discharge Nutritional Support in Malnourished Ill Elderly Patients: Effectiveness and Cost-Effectiveness.

[24] Feldblum I., German L., Castel H., Harman-Boehm I., Shahar D.R. (2011). Individualized nutritional intervention during and after hospitalization: The nutrition intervention study clinical trial. J. Am. Geriatr. Soc..

[25] Buys D.R., Campbell A.D., Godfryd A., Flood K., Kitchin E., Kilgore M.L., Allocca S., Locher J.L. (2017). Meals Enhancing Nutrition After Discharge: Findings from a Pilot Randomized Controlled Trial. J. Acad. Nutr. Diet..

[26] Lindhardt T., Nielsen M.H. (2017). Older patients’ use of technology for a post-discharge nutritional intervention—A mixed-methods feasibility study. Int. J. Med. Infom..

[27] Campbell A.D., Godfryd A., Buys D.R., Locher J.L. (2015). Does Participation in Home-Delivered Meals Programs Improve Outcomes for Older Adults? Results of a Systematic Review. J. Nutr. Gerontol. Geriatr..

[28] Institute of Medicine (2012). Nutrition and Healthy Aging in the Community: Workshop Summary.

[29] Holst M., Rasmussen H.H. (2013). Nutrition therapy in the transition between hospital and home: An investigation of barriers. J. Nutr. Metab..

[30] Coulter A., Ellins J. (2007). Effectiveness of strategies for informing, educating, and involving patients. BMJ.

[31] Baker E.B., Wellman N.S. (2005). Nutrition concerns in discharge planning for older adults: A need for multidisciplinary collaboration. J. Am. Diet. Asso.

[32] Cawood A.L., Elia M., Stratton R.J. (2012). Systematic review and meta-analysis of the effects of high protein oral nutritional supplements. Ageing Res. Rev..

[33] Gazzotti C., Arnaud-Battandier F., Parello M., Farine S., Seidel L., Albert A., Petermans J. (2003). Prevention of malnutrition in older people during and after hospitalisation: Results from a randomised controlled clinical trial. Age Aging.

[34] Stratton R.J., Hébuterne X., Elia M. (2013). A systematic review and meta-analysis of the impact of oral nutritional supplements on hospital readmissions. Ageing Res. Rev..

[35] Zhong Y., Cohen J., Goates S., Luo M., Nelson J., Neumann P. (2017). The Cost-Effectiveness of Oral Nutrition Supplementation for Malnourished Older Hospital Patients. Appl. Health Econ. Health Policy.

[36] Elia M., Normand C., Norman K., Laviano A. (2016). A systematic review of the cost and cost effectiveness of using standard oral nutritional supplements in the hospital setting. Clin. Nutr..

[37] Keller H., McCullough J., Davidson B., Vesnaver E., Laporte M., Gramlich L., Allard J., Bernier P., Duerksen D., Jeejeebhoy K. (2015). The Integrated Nutrition Pathway for Acute Care (INPAC): Building consensus with a modified Delphi. Nutr. J..

[38] Keller H., Laur C., Valaitis R., Bell J., McNicholl T., Ray S., Murphy J., Barnes S. (2017). More-2-Eat: Evaluation protocol of a multi-site implementation of the Integrated Nutrition Pathway for Acute Care. BMC Nutr..

[39] Laporte M., Keller H.H., Payette H., Allard J.P., Duerksen D.R., Bernier P., Jeejeebhoy K., Gramlich L., Davidson B., Vesnaver E. (2015). Validity and reliability of the new Canadian Nutrition Screening Tool in the ‘real-world’ hospital setting. Eur. J. Clin. Nutr..

[40] Detsky A.S., Baker J.P., Johnston N., Whittaker S., Mendelson R.A., Jeejeebhoy K.N. (1987). What is subjective global assessment of nutritional status?. J. Parenter. Enteral Nutr..

[41] Roberts H.C., Denison H.J., Martin H.J., Patel H.P., Syddall H., Cooper C., Sayer A.A. (2011). A review of the measurement of grip strength in clinical and epidemiological studies: Towards a standardised approach. Age Ageing.

[42] McCullough J., Marcus H., Keller H.H. (2017). The Mealtime Audit Tool (MAT)—Inter-rater reliability testing of a novel tool for the monitoring and assessment of food intake barriers in acute care hospital patients. J. Nutr. Health Aging.

[43] McCullough J., Keller H.H. (2016). The My Meal Intake Tool (M-MIT): Validity of a patient self-assessment for food and fluid intake at a single meal. J. Nutr. Health Aging.

[44] Laur C., McCullough J., Davidson B., Keller H.H. (2015). Becoming Food Aware in Hospital: A Narrative Review to Advance the Culture of Nutrition Care in Hospitals. Healthcare.

[45] Kris-Etherton P.M., Akabas S.R., Bales C.W., Bistrian B., Braun L., Edwards M.S., Laur C., Lenders C.M., Levy M.D., Palmer C.A. (2014). The need to advance nutrition education in the training of health care professionals and recommended research to evaluate implementation and effectiveness. Am. J. Clin. Nutr..

[46] Crowley J., O’Connell S., Kavka A., Ball L., Nowson C.A. (2016). Australian general practitioners’ views regarding providing nutrition care: Results of a national survey. Public Health.

